# When place and generation matter: Understanding resident satisfaction in rural tourism

**DOI:** 10.1371/journal.pone.0353554

**Published:** 2026-07-24

**Authors:** Can Zhang, Massoomeh Hedayati Marzbali

**Affiliations:** 1 School of Fine Arts and Art Design, Hunan Institute of Science and Engineering, Yongzhou, Hunan, China; 2 School of Housing, Building and Planning, Universiti Sains Malaysia, Penang, Malaysia; University of Castilla-La Mancha: Universidad de Castilla-La Mancha, SPAIN

## Abstract

The revitalization of traditional villages through rural tourism has emerged as an important pathway for achieving sustainable regional development worldwide. Although the benefits of tourism are widely acknowledged, limited research has explored how residents’ expectations and place-based perceptions influence satisfaction across generations. This study integrates expectation confirmation theory and place attachment theory to explore the influence of expectations, place identity, place attachment, and perceived value on resident satisfaction. Data from 245 residents in three traditional villages in Yongzhou, China, are analyzed using structural equation modeling and multi-group analysis. Results reveal a negative association between expectations and satisfaction, while perceived value mediates the effects of place identity and place attachment. Generational differences further moderate several key relationships, with older residents showing higher satisfaction. These findings deepen our understanding of resident attitudes toward rural tourism and provide practical guidance for inclusive, community-centered strategies that promote sustainable rural development in line with the United Nations Sustainable Development Goals 8, 11, and 12.

## Introduction

Rural areas worldwide are undergoing profound transformations, characterized by economic stagnation, land abandonment, environmental degradation, and demographic shifts such as population aging and decline [1]. These changes pose significant challenges to the livelihoods of rural communities and the preservation of unique cultural landscapes and local heritage. In this context, rural tourism (RT) has emerged as a strategic approach to revitalizing traditional villages by promoting economic growth, cultural continuity, and environmental sustainability, while also enhancing community resilience and long-term social well-being. Its purpose aligns closely with the United Nations Sustainable Development Goals (SDGs), particularly SDG 8 (Decent Work and Economic Growth), SDG 11 (Sustainable Cities and Communities), and SDG 12 (Responsible Consumption and Production) [2].

China provides a particularly striking example of these rural transformations. Between 1991 and 2012, over 1.1 million villages disappeared, leading to an alarming erosion of rural culture and identity [3,4]. Consequently, the diversity of traditional village cultural landscapes (TVCLs)—recognized for their historical, aesthetic, and social value—has been severely diminished [5]. In response, the Chinese government issued the 2018 Guiding Opinions on Promoting Sustainable Rural Tourism, positioning RT as a core revitalization strategy [6].

While RT has contributed to infrastructure upgrading, income diversification, and broader community development, concerns remain regarding cultural commodification, uneven benefit distribution, and the marginalization of residents’ voices [7,8]. Ensuring sustainable outcomes thus requires a deeper understanding of how local residents—who both benefit from and help manage cultural landscapes—perceive and respond to tourism-led changes [9,10]. However, community participation in heritage and tourism management, particularly in rural regions, remains limited [11], highlighting the need to better understand the socio-psychological factors shaping residents’ engagement and satisfaction.

Although residents’ perceptions, attitudes, and support for tourism have been widely studied in the broader tourism literature, significant gaps persist [12,13]. First, generational differences among residents have received insufficient empirical attention in sustainable tourism contexts [14]. Recent research suggests that age, education, income, and generational cohort can significantly influence residents’ environmental attitudes, place perceptions, and satisfaction [14,15]. However, despite growing calls for youth-oriented rural development strategies, comparative studies involving Generation Z remain scarce, even though they differ from older residents in terms of mobility, digital connectedness, future aspirations, and place-based values [16,17]. This gap is even more pronounced in traditional villages marked by the hollow-village phenomenon [18], where sustained youth out-migration has produced an aging resident population and widened generational divides. Such demographic realities underscore the importance of examining differences between Gen Z and non-Gen Z residents. Yet, as Suherlan and Cheer (2024) emphasize, intergenerational perspectives remain insufficiently examined within RT research, despite their relevance for equitable and long-term development [19].

Second, while constructs such as perceived value (PV), community attachment, and sense of place (SoP) have been identified as important determinants of residents’ responses to tourism [20], their interaction with expectation confirmation processes has rarely been examined. Within the framework of expectation confirmation theory (ECT), residents’ expectations serve as cognitive benchmarks against which the functional and experiential outcomes of RT are evaluated, yet ECT has predominantly been applied to tourists or consumers rather than residents [21]. Similarly, place attachment theory (PAT)—particularly through the SoP dimensions of place identity (PI) and place attachment (PA)—offers valuable insights into residents’ emotional and cognitive bonds with their living environment [22]. Integrating ECT and PAT is particularly appropriate for RT contexts, where residents simultaneously assess tourism’s functional benefits, such as livelihood improvements and income diversification, and its implications for place meanings and identity. Despite this conceptual complementarity, integrated models combining ECT, PAT, and PV remain underdeveloped in traditional village tourism research, especially when considering multigenerational differences between Gen Z and non-Gen Z residents [23,24].

To address these gaps, this study investigates residents’ perceptions of tourism development in three traditional villages in Yongzhou, China: Ganyantou, Lanxi, and Shanggantang. Specifically, it explores how expectations, PV, and SoP jointly shape resident satisfaction with RT and assesses whether these relationships vary between Gen Z and non-Gen Z residents. Through integrating ECT and PAT in a multigenerational framework, this study contributes to a deeper theoretical understanding of satisfaction formation in RT contexts and offers evidence-based insights for designing more inclusive, culturally sensitive, and sustainable village revitalization strategies.

## Literature review

### Rural tourism and traditional village cultural landscape

RT refers to tourism activities in non-urban areas that utilize natural landscapes, cultural heritage, and rural facilities to attract visitors seeking authentic rural experiences [8]. TVCLs are outcomes of long-term interactions between human activities and the natural environment, encompassing tangible cultural heritage (e.g., streets, residential spaces, historic structures) and intangible elements (e.g., local customs, social norms, community practices) [25]. These villages have significant historical, cultural, scientific, artistic, social, and economic value, and their preservation is essential for maintaining local identity and heritage [26].

RT in TVCLs often takes the form of cultural heritage tourism, combining tourism activities with rural resources and traditional products to stimulate local economic development, cultural continuity, and community revitalization [27]. Cultural heritage and landscapes serve as key attractions, representing a unique local identity and collective memory [8]. However, tourism development in rural areas is inherently dual in nature, generating opportunities for economic improvement and risks of cultural and environmental transformation [28].

Importantly, the protection and development of traditional villages occur simultaneously, reflecting continuity rather than stagnation [4]. Therefore, sustainable RT requires balancing economic development with the lived experiences and cultural continuity of residents [29]. However, existing research has not sufficiently explained how residents cognitively and emotionally evaluate tourism development outcomes in TVCL contexts, particularly in relation to expectation formation, place-based emotional bonds, and PV creation. This study addresses this gap by integrating cognitive and affective perspectives to explain resident satisfaction with RT development.

### Theoretical foundations: Expectation confirmation theory and place attachment theory

ECT and PAT jointly provide the theoretical foundation for understanding how residents evaluate RT in TVCLs. ECT captures cognitive evaluation processes based on expectation–performance comparisons, whereas PAT explains emotional and symbolic relationships between individuals and places. Although both theories are widely applied in tourism research, they are often treated separately, with limited attention to their joint explanatory power in resident-based RT contexts.

From a theoretical perspective, ECT and PAT originate from different paradigms—cognitive appraisal theory versus affective-symbolic place theory—but jointly explain how residents cognitively evaluate and emotionally interpret tourism development [30,31].

ECT posits that satisfaction results from assessing whether outcomes meet or exceed expectations [32]. Originally developed in consumer behavior [33], ECT has been transferred to various tourism contexts [34,35]. However, most applications conceptualize users as short-term tourists or consumers, whereas residents represent long-term stakeholders embedded in local socio-cultural systems.

In RT contexts, residents’ expectations are not limited to functional or economic improvements but also include emotional and identity-related aspirations, such as continued enjoyment, affection toward place, and preservation of cultural and symbolic landscapes. This extension makes ECT particularly relevant for understanding resident-based evaluations of RT development, where cognitive and affective expectations shape satisfaction formation.

PAT, which originated in environmental psychology, examines the emotional and symbolic bonds individuals develop with places [36]. PA reflects emotional bonds fostering comfort and security, while PI represents the incorporation of place into one’s self-concept [22,37]. Although closely related, PI and PA represent distinct dimensions of place experience: PI is primarily cognitive-symbolic, while PA is affective-emotional.

In this study, PI and PA are conceptualized as interrelated dimensions of SoP, reflecting residents’ cognitive-symbolic identification and affective-emotional bonding with place, respectively. In traditional village contexts, these dimensions are often reinforced through long-term residence, collective memory, kinship networks, and cultural continuity.

Although PAT is widely applied in urban and migration studies [38,39], it remains underexplored in RT research, particularly regarding how different generations—especially Gen Z versus older cohorts—form emotional and cognitive attachments to TVCLs and evaluate tourism development [40].

### Integration of ECT and PAT and the mediating role of perceived value

Integrating ECT and PAT provides a comprehensive framework for understanding residents’ responses to RT development in TVCLs. ECT captures residents’ evaluative cognition based on expectation–performance comparisons, whereas PAT explains how PI and PA—as two interrelated dimensions of SoP—shape residents’ symbolic identification with place and affective-emotional bonds with their living environment.

In traditional villages, residents engage simultaneously in expectation-based evaluations of tourism outcomes and place-based interpretations of tourism-induced transformation. They assess not only whether tourism development delivers anticipated economic and functional benefits, but also whether it aligns with culturally meaningful landscapes, collective memory, community continuity, and identity-related values. Integrating ECT and PAT therefore enables a dual-process explanation of residents’ responses to tourism development by incorporating evaluative and place-based meaning-making processes.

Within this framework, PV is conceptualized as a central cognitive–affective mechanism linking expectations, SoP, and satisfaction. It reflects residents’ overall assessment of the benefits and costs associated with tourism development, incorporating both utilitarian and symbolic dimensions [41,42]. From the perspective of ECT, PV captures residents’ evaluations of whether tourism development meets or exceeds anticipated outcomes and expected benefits. From the perspective of PAT, it reflects the extent to which tourism development supports or threatens emotionally meaningful landscapes, symbolic place meanings, and identity continuity.

Accordingly, PV serves as a bridging mechanism through which residents translate expectation-based evaluations and place-based emotional meanings into overall satisfaction judgments. Although PV has been widely examined in service quality and satisfaction models such as the American Customer Satisfaction Index (ACSI) [33], prior RT studies rarely examine it as a mediating mechanism linking expectations, SoP, and satisfaction [41,42]. Existing studies primarily focus on direct relationships among these constructs, leaving the underlying integrative mechanism insufficiently theorized.

Furthermore, the application of generational cohort theory suggests that the evaluative mechanisms underlying RT perceptions may not be uniform across residents. Individuals shaped by different socio-cultural and technological environments develop distinct value formation logics and interpretive frameworks [43]. Gen Z residents tend to construct PV through experiential and symbolic interpretations of RT [16], whereas older residents rely more strongly on long-term PA and identity continuity when evaluating tourism outcomes [44]. These differences suggest that generational background conditions how expectation-based and place-based evaluations are translated into PV and satisfaction.

To address these gaps, this study positions PV as a central mediating construct that connects expectation-based evaluations and place-based symbolic and emotional meanings in shaping resident satisfaction with RT development in TVCLs. Overall, ECT and PAT jointly inform a unified structural model in which expectation-based cognitive evaluations and place-based affective meanings converge through PV to shape resident satisfaction, with generational differences further moderating these relationships.

### Hypothesis development

#### Cognitive influences on perceived value and satisfaction.

PV reflects residents’ trade-off between benefits and costs, integrating both functional and emotional elements [45–47]. Within ECT, expectations guide subsequent evaluations by serving as cognitive benchmarks for interpreting performance outcomes [32]. Prior tourism studies confirm that expectation–performance comparisons shape value perceptions for both tourists and local residents [48,49]. Local studies similarly report that residents’ expectations about tourism development directly affect their perceived impacts [50]. When residents perceive that tourism development aligns with anticipated economic or social benefits, their PV tends to increase [51].

Empirical findings on the expectations–PV relationship remains mixed. While some studies document positive links between expectations and satisfaction [52,53], others find non-significant or negative relationships [54], particularly in early-stage or underdeveloped tourism contexts where high expectations lead to negative disconfirmation [42]. Expectations may also exert direct and indirect effects on satisfaction through PV. To address this complexity, the following hypotheses are proposed:

**H1**: Residents’ expectations positively influence their perceived value of TVCLs in rural tourism development.

**H2**: Residents’ expectations negatively influence their satisfaction with TVCLs in rural tourism development.

#### Emotional influences on perceived value and satisfaction.

SoP—conceptualized through PI and PA—plays a critical role in how residents evaluate tourism impacts. Consistent with PAT, PI is considered to reinforce PA by strengthening emotional bonds through identity-based meaning construction [55].

SoP has been widely recognized as both an antecedent and an outcome in tourism research. While earlier studies primarily treated it as an outcome of PV among tourists [56], more recent evidence suggests that SoP may also function as an antecedent for residents in shaping value perceptions [24]. Strong emotional bonds enhance residents’ valuation of tourism benefits, especially in areas facing depopulation and economic decline. PI further reinforces PA, providing an emotional foundation for tourism-related evaluations [57].

SoP is also closely associated with satisfaction, defined as residents’ overall appraisal of tourism’s effects on their environment and quality of life [58,59]. However, satisfaction may also strengthen SoP over time [60], indicating possible reciprocity. In traditional villages, strong SoP can buffer or amplify reactions to tourism-induced changes. Residents with higher PI or PA may perceive greater value yet experience lower satisfaction if development disrupts valued landscapes. Based on these insights, this study hypothesizes the following:

**H3**: Place identity positively influences perceived value of TVCLs in rural tourism development.

**H4**: Place attachment positively influences perceived value of TVCLs in rural tourism development.

**H5**: Place identity positively influences place attachment toward TVCLs in rural tourism development.

**H6**: Place identity positively influences satisfaction toward TVCLs in rural tourism development.

**H7**: Place attachment positively influences satisfaction toward TVCLs in rural tourism development.

#### Integrated mechanisms and mediating role of perceived value.

Integrating ECT and PAT provides a dual-process framework for understanding residents’ responses to RT development in TVCLs. Within ECT, PV mediates the link between expectations and satisfaction by reflecting the perceived alignment between anticipated and actual outcomes [61]. PV has been widely examined in tourist evaluations, such as in traditional villages, industrial heritage sites, and creative destinations [62–64]. However, it has not been empirically verified in rural communities, where residents’ expectations relate to the functional improvements brought by RT and the preservation, aesthetic quality, and experiential attributes of TVCLs.

Beyond this cognitive pathway, PAT suggests that emotional ties such as PI and PA influence how residents assess the value of tourism. While SoP components have been associated with satisfaction [65], the possibility that PI and PA shape satisfaction through PV has rarely been tested. Emerging evidence hints at such mechanisms: PA affects residents’ support for tourism [24], and PI influences behavioral intentions via PV [66]. These insights indicate that PV may serve as a cognitive–emotional bridge linking expectations, place-based emotions, and satisfaction. This study empirically evaluates these mediating pathways, thereby addressing a notable gap in RT and resident-focused research. Building on these insights, this study proposes the following hypotheses:

**H8**: Perceived value positively influences satisfaction toward TVCLs in rural tourism development.

**H9**: Perceived value mediates the relationship between expectations and satisfaction.

**H10:** Perceived value mediates the relationship between place identity and satisfaction.

**H11**: Perceived value mediates the relationship between place attachment and satisfaction.

#### Moderating role of generational differences.

Generational identity offers a critical lens for understanding whether the core evaluative mechanism in RT—from SoP to PV to satisfaction—functions uniformly across residents. Individuals from different generational groups have been shaped by distinct socio-cultural and technological environments that influence how they interpret and evaluate place-based experiences [67–69].

In particular, differences between Gen Z and older cohorts are theoretically meaningful in RT contexts. Older residents tend to develop stronger PI and PA due to long-term residency and cultural continuity [44,70]. However, their evaluations of tourism impacts remain mixed: while some appreciate its potential benefits [71], others are more concerned about social change and cultural disruption [72,73]. These emotional responses may shape how SoP translates into value perceptions.

Conversely, younger cohorts—especially Gen Z (born 1995–2009)—tend to form weaker local attachment but show higher sensitivity to aesthetic, experiential, and symbolic qualities in rural landscapes [16]. They also evaluate heritage spaces differently [74]. Such differences suggest that generations may vary not only in their emotional bonds (SoP) but also in how these bonds are cognitively converted into PV.

Despite the abundance of tourist-focused studies on Gen Z, research comparing Gen Z to non-Gen Z residents remains scarce. Most resident studies contrast “young vs. old,” without adopting generational theory as an analytical framework. This gap is notable given that Gen Z is becoming a dominant cultural force in China, yet often has weaker local attachment and different value orientations [75,76]. A generational comparison can reveal whether satisfaction formation processes differ across residents shaped by different socio-cultural eras [77,78].

Understanding generational moderation therefore helps determine whether emotional attachment (SoP) leads to value assessments (PV) and satisfaction in the same way across different cohorts, or whether these pathways vary for residents shaped by different socio-cultural eras. Accordingly, this study proposes the following hypotheses:

**H12**: The relationship between place identity and place attachment differs across generations.

**H13**: The relationship between place identity and perceived value differs across generations.

**H14**: The relationship between place attachment and perceived value differs across generations.

**H15**: The relationship between place identity and satisfaction differs across generations.

**H16**: The relationship between place attachment and satisfaction differs across generations.

**Fig 1:**
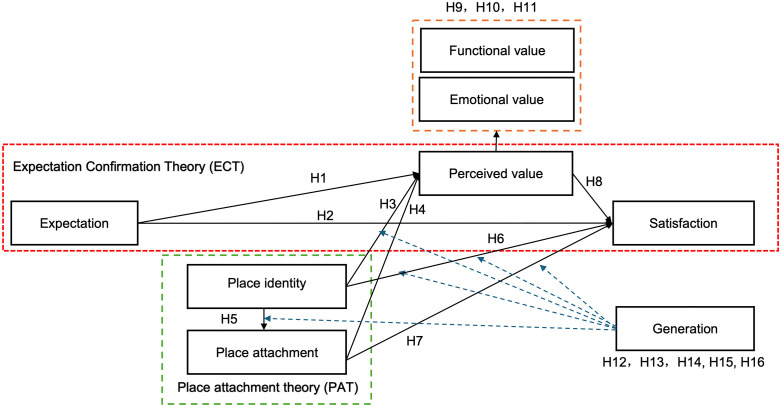
Conceptual framework.

Fig 1 presents the research model for this study, drawn from the aforementioned literature and conceptual assumptions.

## Methods

### Study area

The preservation and revitalization of traditional villages have become national priorities in China, with RT increasingly promoted as a mechanism for cultural landscape protection and rural development. Yongzhou, a nationally designated historical and cultural city in Hunan Province, exemplifies this trajectory. As of 2023, Yongzhou had 96 officially recognized traditional villages. This study focuses on three representative sites classified as Chinese Historical and Cultural Villages, namely, Ganyantou, Shanggantang, and Lanxi [79]. These villages are distinguished by well-preserved Ming–Qing architectural ensembles and rich intangible cultural heritage, including traditional festivals, crafts, and long-standing local customs, making them suitable for examining resident perceptions of tourism within TVCLs.

In addition to their cultural significance, these villages reflect the socio-economic characteristics typical of many TVCLs. Compared with national and provincial averages, Yongzhou remains less developed, with rural residents accounting for 75.1% of the total population and exhibiting accelerated population aging due to the sustained outmigration of younger cohorts seeking employment elsewhere [80] Consequently, the demographic structure of these villages is characterized by the predominance of older long-term residents and the relatively transient presence of younger generations, particularly those born after 1995.

National assessments further indicate the vulnerability of traditional villages, with 44.6% of active villages in the Yangtze and Yellow River basins disappearing between 2010 and 2014 due to economic decline, outmigration, and weakening community structures [81]. Yongzhou displays similar patterns of cultural richness juxtaposed with economic marginality and demographic hollowing. These characteristics render the selected villages appropriate for investigating resident evaluations of RT and comparing generational differences—especially between Gen Z and non–Gen Z residents—in expectations, value perceptions, and cultural attachment within TVCLs.

### Instrument development

The measurement framework was developed based on established scales in the tourism and consumer satisfaction literature. Resident expectations were conceptualized using items adapted from Hui et al. (2007) [82], focusing on anticipations regarding tourism outcomes.

PV was treated as a 2-D second-order reflective construct comprising emotional and functional value. Emotional value items were based on Sweeney and Soutar (2001) [83] and Zhang et al. (2022) [84], while functional value items drew from Eid and El-Gohary (2015) [85] and Sato et al. (2018) [86]. The higher-order construct of PV was modeled in SmartPLS using the repeated indicator approach, in which all indicators of the lower-order constructs were assigned to the higher-order construct.

SoP served as an overarching conceptual framework in this study. However, PA and PI were operationalized as two distinct but theoretically related constructs and examined separately in the structural model. PA items were informed by Gross and Brown (2006) and Jorgensen and Stedman (2006) [87,88], and PI items by Gross and Brown (2006) and Zhao et al. (2022) [88,89].

Resident satisfaction was captured through overall satisfaction, drawing from Bruwer (2014) and Park et al. (2019) [90,91]. All constructs were measured using a five-point Likert scale (1 = strongly disagree to 5 = strongly agree).

Partial least squares structural equation modeling (PLS-SEM) was employed to assess the proposed model. PLS-SEM is appropriate for exploratory research, particularly when dealing with complex models and non-normal data distributions and when the sample size is relatively small or imbalanced [92]. In this study, two factors influenced the selection of PLS-SEM: (1) the unequal distribution of Gen Z and non-Gen Z residents within the sample, and (2) the analytical strength of PLS-SEM in handling formative and reflective constructs. Robustness was ensured using a bootstrapping procedure with 10,000 resamples [93].

### Sampling and data collection

This study focused on residents aged 18–65 years from three RT villages in China: Ganyantou, Lanxi, and Shanggantang. Participants were prospectively recruited between July and October 2024 using convenience sampling. Surveys were administered both face-to-face and online in residential areas, village squares, tourist sites, and public parks.

The study protocol was reviewed and approved by the Ethics Committee of Hunan University of Science and Engineering. Participation was entirely voluntary, and verbal informed consent was obtained from all participants prior to data collection and documented in accordance with the procedures approved by the Ethics Committee. Respondents completed self-administered questionnaires, with assistance provided when necessary.

A pre-test involving 30 randomly selected residents was conducted to evaluate the questionnaire’s psychometric properties and ensure clarity and reliability. Feedback from the pre-test prompted minor revisions in wording for improved comprehension. Sample size requirements were calculated using G*Power 3.1. For a t-test with a medium effect size (d = 0.5), an alpha level of 0.05, and statistical power of 0.80, a minimum of 51 participants per group was needed [94]. In the formal survey, 300 questionnaires were distributed equally across the three villages, yielding an overall response rate of 81.6%.

The sample reflects demographic characteristics common to many rural Chinese communities, including high youth outmigration and relatively low educational attainment among residents. These factors contributed to a lower online response rate and a survey process that sometimes required direct assistance, particularly for older adults. Despite these challenges, 90 valid responses were obtained from Gen Z participants (ages 18–29), enabling generational subgroup analysis. The final sample met the minimum statistical requirements for the power analysis, enabling valid intergenerational comparisons in the subsequent analysis.

## Data analysis and result

### Preliminary analysis

A total of 245 valid responses were collected. Female participants (53.1%, n = 130) slightly outnumbered male participants (46.9%, n = 115). Age distribution was relatively balanced, with 36.7% (n = 90) aged 18–29 and 63.3% (n = 155) aged 30 or above.

Educational attainment was generally low: 75.5% (n = 185) had completed high school or less, and none held a postgraduate degree. Correspondingly, 75.5% reported a monthly household income below 3,000 Yuan, reflecting limited economic resources in the communities.

Regarding tourism-related behavior, 72.2% of respondents reported infrequent travel, and 60.8% had limited interaction with tourists. Most relied on word-of-mouth (64.9%) for destination information, followed by online platforms (20.0%) and travel agencies (11.4%). These results suggest a strong dependence on interpersonal networks and limited exposure to formal tourism promotion.

### Multicollinearity

Multicollinearity, referring to high correlations among predictor constructs that may distort parameter estimates, was assessed using variance inflation factor (VIF) values following Hair et al. (2014) [92]. Analysis using SmartPLS 4.0 revealed outer VIF values ranging from 1.374 to 3.286 (Table 1), all below the recommended threshold of 3.3, indicating that indicator-level multicollinearity exists and is within acceptable limit [95]. Given the conceptual proximity between PI and PA within the SoP framework, a moderate degree of collinearity between these constructs is expected and is further supported by satisfactory discriminant validity (heterotrait–monotrait ratio [HTMT] < 0.90).

**Table 1:** Measurement model for the first-order constructs.

Constructs	Indicators	Outer Loading	Cronbach’s alpha	CR	AVE	VIF
**Expectation**	EX01	0.834	0.780	0.855	0.598	1.636
	EX02	0.830				1.661
	EX03	0.762				1.638
	EX04	0.654				1.374
**Place attachment**	PA01	0.813	0.858	0.904	0.702	1.839
	PA02	0.841				2.193
	PA03	0.840				1.977
	PA04	0.856				2.293
**Place identity**	PI01	0.879	0.910	0.937	0.788	2.873
	PI02	0.910				3.286
	PI03	0.901				3.104
	PI04	0.861				2.384
**Emotional value**	EV01	0.880	0.887	0.917	0.689	2.737
	EV02	0.814				2.109
	EV03	0.814				2.124
	EV04	0.819				2.213
	EV05	0.820				2.273
**Functional value**	QFV01	0.874	0.890	0.924	0.752	2.582
	QFV02	0.869				2.516
	QFV03	0.840				2.097
	QFV04	0.886				2.597
**Overall satisfaction**	SA01	0.816	0.861	0.906	0.706	1.733
	SA02	0.837				2.083
	SA03	0.849				2.128
	SA04	0.858				2.227

Construct-level (inner) VIF values were also assessed (Table 3). All inner VIF values ranged from 1.000 to 3.180, remaining below the recommended threshold of 3.3, suggesting that structural collinearity does not bias the estimated path coefficients.

### Common method bias

As all data were collected through self-reported questionnaires at a single point in time, common method bias (CMB) was assessed using Harman’s single-factor test. All measurement items were entered into an unrotated principal component analysis. The first unrotated factor accounted for 47.59% of the total variance, below the commonly accepted threshold of 50% in behavioral and management research [96]. Therefore, CMB was unlikely to pose a serious threat to the validity of the findings.

### Measurement model assessment

Prior to factor analysis, the Kaiser–Meyer–Olkin (KMO) measure and Bartlett’s test of sphericity were conducted to assess sampling adequacy and the suitability of the data. The KMO value was 0.944, and Bartlett’s test was significant (p < 0.001), indicating that the dataset was well-suited for factor analysis [97].

In this study, SmartPLS 4.0 was used to assess the reliability and validity of the measurement model (Table 1). Reliability was evaluated through standardized factor loadings, Cronbach’s alpha (α), and composite reliability (CR), indicating strong internal consistency. Factor loadings ranged from 0.654 to 0.886, with the lowest for item Expectation 04 (0.654), exceeding the acceptable threshold of 0.40 [98]. Cronbach’s α ranged from 0.780 to 0.910, and CR from 0.855 to 0.937, all above the recommended 0.70 level [92]. Convergent validity was supported by AVE values between 0.598 and 0.788, surpassing the 0.50 criterion [92]. These results confirm the reliability and validity of the measurement model, ensuring a solid basis for further analysis.

Given the tendency of PLS-SEM to inflate AVE and underestimate inter-construct correlations, discriminant validity was further assessed using the HTMT ratio [99]. As shown in Table 2, all HTMT values were below the conservative threshold of 0.90, confirming adequate discriminant validity.

**Table 2:** Discriminant validity-HTMT matrix.

	ᵃEV	^b^EX	PA	PI	^c^QFV	^d^SA
**EV**						
**EX**	0.524					
**PA**	0.799	0.601				
**PI**	0.788	0.424	0.846			
**QFV**	0.701	0.387	0.742	0.619		
**SA**	0.784	0.371	0.801	0.708	0.871	

ᵃEV = emotional value; ᵇEX = expectation; ^c^QFV = quality functional value; ^d^SA = overall satisfaction.

### Structural model assessment

**Fig 2:**
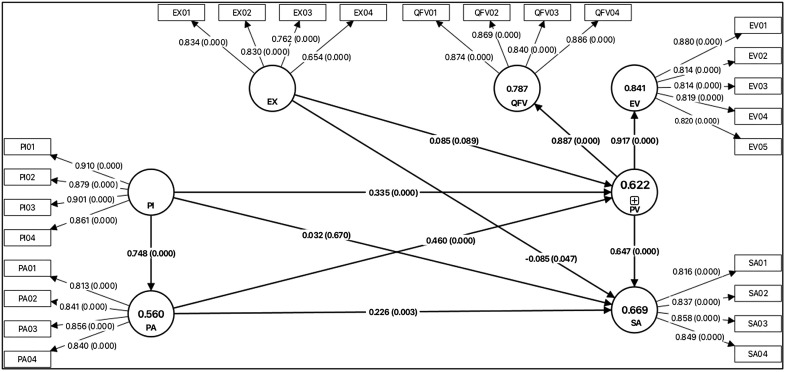
Estimates from the structural model.

Model fit was assessed using SmartPLS. The standardized root mean square residual value was 0.099, below the 0.10 threshold, indicating acceptable fit [100]. As shown in Fig 2, the inner model presents path coefficients and significance levels, while the outer model displays indicator loadings and p-values. R^2^ values ranged from 0.560 to 0.841, suggesting moderate to high explanatory power [101]. Q^2^ values ranged from 0.375 to 0.587, demonstrating medium to strong predictive relevance and supporting overall model adequacy [92].

To test the statistical significance of model paths, a bootstrapping procedure with 10,000 resamples was conducted in SmartPLS. Bias-corrected confidence intervals (CIs; 97.5%) were used to evaluate the direct and indirect effects. A two-tailed t-value above 1.96 (p < 0.05) was considered statistically significant, with critical values of 2.576 and 1.645 corresponding to 1% and 10% significance levels, respectively [102]. The results of hypothesis testing are summarized in Tables 3 and 4 and visually depicted in Fig 2.

**Table 3:** Path coefficients and inner VIF.

Path Coefficients Analysis											
Hypothetical		F^2^	β	M	STDEV	T	P	2.5%	97.5%	VIF	Supported
**H1**	EX - > PV	0.014	0.085	0.088	0.050	1.703	0.089**	−0.017	0.180	1.341	NO
**H2**	EX - > SA	0.016	−0.085	−0.082	0.043	1.990	0.047**	−0.170	−0.001	1.360	YES
**H3**	PI - > PV	0.131	0.335	0.331	0.076	4.430	0.000***	0.171	0.467	2.271	YES
**H4**	PA - > PV	0.214	0.460	0.461	0.072	6.391	0.000***	0.323	0.605	2.619	YES
**H5**	PI - > PA	1.271	0.748	0.749	0.047	15.863	0.000***	0.638	0.824	1.000	YES
**H6**	PA - > SA	0.049	0.226	0.225	0.075	3.007	0.003***	0.090	0.389	3.180	YES
**H7**	PI - > SA	0.001	0.032	0.031	0.075	0.426	0.670	−0.122	0.170	2.567	NO
**H8**	PV - > SA	0.478	0.647	0.648	0.062	10.441	0.000***	0.519	0.765	2.648	YES
**Specific Indirect Effects**											
**Hypothetical**			**β**	**M**	**STDEV**	**T**	**P**	**2.5%**	**97.5%**	**Supported**	
**H9**	EX - > PV - > SA		0.055	0.057	0.033	1.664	0.090*	−0.007	0.124	NO	
**H10**	PI - > PV - > SA		0.298	0.299	0.054	5.566	0.000***	0.107	0.323	YES	
**H11**	PA - > PV - > SA		0.217	0.215	0.054	3.988	0.000***	0.199	0.408	YES	

*p ＜ 0.1 when t-value ≥1.645 two-tail test, **p ＜ 0.05 when t-value ≥1.960 two-tail test, ***p ＜ 0.01 when t-value ≥2.576 two-tail test.

**Table 4:** Mediating variable analysis.

IV	Mediator	DV	Direct effect(O/T)	Indirect effect(O/T)	Total effect	VAF	Strength Rating
PI	PV	SA	0.032 (0.426)	0.217 (3.988)	0.640	33.90%	Partial
PA	PV	SA	0.226 (3.007)	0.298 (5.566)	0.524	56.87%	Partial

#### Test of direct effects.

Table 3 presents the analysis of direct effects, revealing several statistically significant relationships: PI → PV (β = 0.335, *p* = 0.000, *t* = 4.430), PA → PV (β = 0.460, *p* = 0.000, *t* = 6.391), PI → PA (β = 0.748, *p* = 0.000, *t* = 15.863), PA → SA (β = 0.226, *p* = 0.003, *t* = 3.007), and PV → SA (β = 0.647, *p* = 0.000, *t* = 10.441). All these paths exhibit *p*-values below 0.05, positive β-values, and *t*-values exceeding the threshold of 1.96, indicating strong statistical significance.

Notably, the path from EX → SA (β = –0.085, *p* = 0.047, *t* = 1.990) is also statistically significant. Although the effect is negative, it aligns with the hypothesized direction. However, the paths from EX → PV (β = 0.085, *p* = 0.089, *t* = 1.703) and PI → SA (β = 0.032, *p* = 0.670, *t* = 0.426) are not statistically significant, as their *p*-values exceed 0.05 and *t*-values fall below 1.96.

Overall, these findings provide empirical support for H2, H3, H4, H5, H6, and H8.

#### Test of mediation effect.

Table 3 presents the analysis of mediation effect, revealing significant indirect effects for the pathways PI → PV → SA (β = 0.289, *p* = 0.000, *t* = 5.566) and PA → PV → SA (β = 0.217, *p* = 0.000, *t* = 3.988), thus providing empirical support for H10 and H11. By contrast, the indirect effect of EX → PV → SA (β = 0.055, *p* = 0.090, *t* = 1.664, CI = [−0.007, 0.124]) was not statistically significant, as the CI included 0. Therefore, H9 is not supported.

Table 4 presents the variance accounted for (VAF) values, which assess the extent of mediation effects within the model. A VAF greater than 80% indicates full mediation, values between 20% and 80% suggest partial mediation, and values below 20% imply no mediation [103]. The results indicate that PV partially mediates the relationship between PI and satisfaction (VAF = 33.90%), as well as between PA and satisfaction (VAF = 56.87%).

#### Test of moderation effects.

An independent samples t-test was conducted to compare Gen Z (≤29 years old, n = 90) and non-Gen Z residents (≥30 years old, n = 155) with respect to PA, PI, PV, and satisfaction. Results indicated that Gen Z participants reported significantly lower scores across all constructs (Table 5), with the largest differences in PI and PA, suggesting that younger residents exhibit weaker emotional and identity-based connection to the RT context.

**Table 5:** Independent sample t-tests of age.

Constructs	group	Mean	Std. Deviation	Std. Error Mean	t	df	Sig. (2-tailed)	Mean Difference	Std.Error Difference	95% CI	
**Lower**	**Upper**
**PA**	^a^1	4.030	0.831	0.088	−6.070	120.639	0.000***	−0.576	0.095	−0.764	−0.388
	^b^2	4.606	0.455	0.037							
**PI**	^a^1	3.922	0.923	0.097	−5.615	128.771	0.000***	−0.603	0.107	−0.816	−0.391
	^b^2	4.525	0.567	0.046							
**PV**	^a^1	4.088	0.760	0.080	−3.771	135.197	0.000***	−0.339	0.090	−0.516	−0.161
	^b^2	4.427	0.504	0.041							
**SA**	^a^1	4.098	0.795	0.084	−2.644	142.653	0.009***	−0.252	0.095	−0.441	−0.064
	^b^2	4.350	0.569	0.046							

^a^1 = Z generation group (90 cases)；^b^2 = non-Z generation group (155 cases).

Before conducting multi-group analysis (MGA), the measurement invariance of composite models (MICOM) procedure was performed following Henseler et al. (2016) to assess measurement invariance between the Gen Z and non-Gen Z groups [104]. First, configural invariance was established because both groups used identical indicators, data treatment procedures, and algorithm settings. Second, compositional invariance was assessed using the permutation procedure. The results indicated compositional invariance for three of the four constructs (Table 6). For PA, the original correlation (0.997) fell marginally below the 5% quantile (0.998), representing a negligible deviation of only 0.001. Given that all other constructs satisfied the compositional invariance criterion and the deviation for PA was substantively trivial, partial measurement invariance was considered established. Therefore, meaningful comparisons of path coefficients across groups through MGA were deemed appropriate [104].

**Table 6:** MICOM compositional invariance assessment.

	Original correlation	Correlation permutation mean	5.00%	Permutation p value
**PA**	0.997	0.999	0.998	0.020
**PI**	1.000	1.000	0.999	0.774
**PV**	1.000	0.999	0.999	0.440
**SA**	1.000	0.999	0.998	0.523

MGA was conducted to further explore generational differences (Table 7). The relationship between PA and PV was positive and significant for both Gen Z (β = 0.440, p < 0.001) and non-Gen Z respondents (β = 0.423, p < 0.001), with no significant difference observed between the two groups (Δβ = 0.017, p = 0.888). Similarly, PI positively influenced PV among both Gen Z (β = 0.412, p < 0.001) and non-Gen Z respondents (β = 0.257, p = 0.029), but the intergroup difference was not statistically significant (Δβ = 0.155, p = 0.333). In addition, the relationship between PA and satisfaction was positive and significant for both Gen Z (β = 0.359, p = 0.002) and non-Gen Z respondents (β = 0.152, p = 0.050), with no significant difference across generations (Δβ = 0.207, p = 0.139). Therefore, H13, H14, and H16 are not supported.

**Table 7:** Multi-group analysis of age.

Hypothesis		Group	Difference (1–2）	2-tailed (1 vs 2) p value	Original	2.50%	97.50%	P	T	Supports
**H12**	PI - > PA	^a^1	0.251	0.007	0.797	0.686	0.866	0.000	17.886	YES
		^b^2			0.546	0.384	0.698	0.000	6.581	
**H13**	PI - > PV	^a^1	0.155	0.333	0.412	0.203	0.631	0.000	3.789	NO
		^b^2			0.257	−0.001	0.459	0.029	2.185	
**H14**	PA - > PV	^a^1	0.017	0.888	0.440	0.210	0.646	0.000	3.954	NO
		^b^2			0.423	0.253	0.584	0.000	5.027	
**H15**	PI - > SA	^a^1	−0.278	0.049	−0.129	−0.376	0.105	0.295	1.048	YES
		^b^2			0.150	0.013	0.291	0.033	2.130	
**H16**	PA - > SA	^a^1	0.207	0.139	0.359	0.132	0.587	0.002	3.109	NO
		^b^2			0.152	0.001	0.307	0.050	1.960	

However, significant generational moderation was found for two hypotheses. For H12 (PI → PA), the path was significant in both groups but was stronger among Gen Z respondents (β = 0.797, p < 0.001) than among non-Gen Z respondents (β = 0.546, p < 0.001). The difference between the two groups was statistically significant (Δβ = 0.251, p = 0.007), thereby supporting H12.

For H15 (PI → satisfaction), the path was not significant for Gen Z respondents (β = −0.129, p = 0.295) but was positive and significant for non-Gen Z respondents (β = 0.150, p = 0.033). A significant intergroup difference was observed (Δβ = −0.278, p = 0.049), supporting H15.

## Discussion and implications

### Main findings

This study investigated the complex relationships among residents’ expectations, SoP (including PA and PI), PV (emotional and functional), and satisfaction within the context of RT development in TVCLs. Using PLS-SEM and MGA, several notable findings emerged.

First, consistent with ECT, residents’ expectations had a significant negative effect on satisfaction, indicating a gap between anticipated and actual outcomes of RT development. This finding challenges the conventional assumption of a positive linear relationship proposed in the ACSI model [33] and indicates that inflated or idealized expectations may generate disconfirmation, thereby reducing satisfaction. One plausible explanation is that long-term residents tend to assess tourism benefits through livelihood and authenticity considerations [21,105], making them more sensitive to unmet expectations.

Second, PI had no direct effect on satisfaction across the full sample but was significant among non-Gen Z residents. This generational divergence implies that younger residents construct PI more symbolically and experientially, linking it indirectly to satisfaction through value perceptions. By contrast, older residents—whose identity is shaped by nostalgia and long-term belonging [106]—maintain a more direct connection between PI and satisfaction.

Third, PV partially mediated the effects of PI and PA on satisfaction, confirming its pivotal role as a cognitive–affective bridge in residents’ evaluations. Although MGA did not reveal statistically significant generational differences in the PI–value path, further subgroup inspection revealed that this relationship was stronger for Gen Z. Exposed to visually mediated and digitally enriched environments [16], younger residents may be more responsive to identity cues embedded in local landscapes. Older residents, by contrast, draw on habitual familiarity and continuity, which shape satisfaction more directly than value assessment.

The effects of PA on PV and satisfaction were robust and significant across generations as well. This underscores the universal role of attachment in shaping emotional and cognitive evaluations of RT development [24]. Finally, the study confirmed a significant positive relationship between PI and PA, consistent with prior research [57]. Interestingly, despite older residents scoring higher in both constructs, the structural link from identity to attachment was stronger among Gen Z, suggesting a more dynamic, experience-based identity formation process among younger cohorts.

### Theoretical implications

This study advances theoretical understanding in several ways.

First, it extends ECT into a resident-centered, community context. While ECT typically posits that higher expectations enhance satisfaction through confirmation [61], our findings reveal an inverse relationship among long-term rural residents. This recontextualization highlights that, in public or collective settings—unlike consumer contexts—expectations may amplify perceived gaps between idealized outcomes and actual experiences. It therefore supports calls for situating ECT within socio-cultural frameworks that acknowledge context-sensitive expectation dynamics [107].

Second, this study extends the scope of PAT by situating PA and PI within the lived experiences of rural communities, a group often overlooked in mainstream tourism literature [40]. While much prior research has emphasized how PV influences PA [56,108], our findings reverse this causal direction, showing that both PA and PI significantly shape residents’ PV. This aligns with and builds on emerging work [24] that reconceptualizes value as a product of emotional and symbolic place bonds rather than just utility-based judgments.

Another key theoretical contribution lies in identifying PV as a central mediating construct between SoP and satisfaction. This highlights the cognitive-emotional integration process by which residents evaluate RT development, reinforcing the idea that affective and identity-based constructs can inform rational judgments of value and satisfaction. Such insights help to bridge the theoretical gap between place-based emotional theories (e.g., PAT) and expectation-based cognitive frameworks (e.g., ECT).

Lastly, by incorporating generational perspectives, this study introduces a novel analytical lens into PAT and ECT applications. While Gen Z has gained increasing attention as a tourism consumer group, their role as residents and stakeholders in cultural landscape governance remains largely unexamined [109]. Our study reveals distinct generational patterns: although both groups demonstrate strong PA contributing to high PV and satisfaction, their perceptions of PI, its influence on PA and PV, and its contribution to satisfaction vary significantly. These generational differences provide a concise theoretical and practical basis for tailoring RT strategies to different age groups.

### Managerial implications

The findings of this study offer several actionable insights for policymakers and planners promoting sustainable RT, thereby contributing directly to SDGs 8, 11, and 12.

First, managing residents’ expectations is crucial, particularly for long-term residents who are sensitive to gaps between anticipated and actual outcomes. Local governments can establish regular participatory mechanisms, such as quarterly community meetings and satisfaction surveys, while considering residents’ different roles (e.g., guides, vendors, or general households). Clear communication of project objectives, realistic timelines, and transparent benefit-sharing mechanisms can narrow expectation–outcome gaps and strengthen residents’ trust in tourism initiatives.

Second, enhancing functional and emotional value should be tailored by age group. For older residents, planners can prioritize infrastructure upgrades, heritage building restoration, and stable tourism-related employment, while reinforcing emotional value through village storytelling sessions and local history exhibitions. For younger residents, digital platforms such as short video storytelling, online heritage mapping, and co-creation workshops can actively engage them in shaping village identity and fostering long-term attachment. Such initiatives also help convey local culture effectively beyond geographical limitations, as illustrated by “the 81 ‘Hes’ of Shanggangtang.”

Furthermore, institutionalizing inclusive and generationally sensitive participation can enhance satisfaction and PV. Establishing youth advisory councils, intergenerational planning committees, or co-creation teams encourages meaningful engagement from all age groups. Activities such as intergenerational cultural debates, digital–traditional creative competitions, collaborative arts projects, or community-led entrepreneurship initiatives can increase overall participation, reduce generational gaps, and strengthen community cohesion.

Finally, implementing these strategies in an integrated manner can transform residents from passive beneficiaries into active co-creators of RT development. Clear monitoring and evaluation mechanisms, such as participation tracking, resident satisfaction surveys, and economic indicators, allow policymakers to assess effectiveness and adjust interventions over time, supporting culturally sensitive and sustainable rural development consistent with national and international policy guidance.

## Conclusion

This study examined how expectations, SoP, and PV jointly shape resident satisfaction in RT within TVCLs. By integrating ECT and PAT, it highlights PV as a key cognitive–affective mechanism that translates emotional and identity-based bonds into satisfaction outcomes [24,33,61].

The negative relationship between expectations and satisfaction challenges conventional consumer-oriented assumptions and emphasizes the contextual distinctiveness of community-based tourism. In less-developed areas, residents’ expectations for cultural landscapes often exceed their perceived satisfaction, particularly in socially complex and culturally sensitive communities, highlighting the importance of understanding expectations in a phased and layered manner [42].

Generational differences further reveal evolving modes of engagement with place. The identity and attachment of older residents are rooted in long-term belonging and nostalgia, whereas younger residents exhibit more fluid, symbolically oriented relationships shaped by digital connectivity and cultural reinterpretation [16,74]. These differences suggest that strategies should account for functional and experiential differences while also fostering overall community cohesion to align generational perspectives.

Theoretically, this study extends ECT and PAT by integrating cognitive expectations, emotional bonds, and evaluative outcomes within a community context while empirically illustrating generational variability. However, these mechanisms are likely most applicable to villages that are either historically and culturally rich or economically underdeveloped. In more urbanized or economically advanced settings, expectation–satisfaction dynamics and the role of PV may differ, warranting caution in generalization.

Practically, the findings support policies that balance functional development with emotional and symbolic enrichment, enhance PV through participatory governance, and promote intergenerational cohesion. Treating residents as co-creators rather than passive beneficiaries is crucial for achieving satisfaction and sustainable rural revitalization. These strategies also contribute to the United Nations SDGs 8, 11, and 12 and are reflected in China’s 2018 Guiding Opinions on Promoting Sustainable Rural Tourism, ensuring traditional villages remain vibrant, meaningful, and inclusive in a rapidly changing socio-economic landscape.

### Limitations and future research

This study has several limitations that warrant consideration. First, the sample is drawn from villages experiencing the “hollowing-out” phenomenon, resulting in the underrepresentation of Gen Z residents. While statistically adequate, this generational imbalance may limit the generalizability of multi-group comparisons. The unequal subgroup sizes may also lead to larger standard errors and lower statistical power in the MGA, potentially weakening the detection of significant intergroup differences. Therefore, the generational comparison results should be interpreted with caution. Future research should improve youth representation by expanding geographic coverage or targeting migrant populations through digital platforms. Second, the cross-sectional design restricts insights into temporal dynamics. Longitudinal studies are needed to capture how PI, PA, PV, and satisfaction evolve over time under changing socio-economic conditions. Third, the findings are context-specific and may not extend to other regions or cultural settings. Comparative studies across diverse RT contexts could assess the cross-cultural robustness of the model and identify contextual moderators. Lastly, future work should integrate qualitative methods to explore the subjective meanings and lived experiences underlying the observed statistical relationships.

## Supporting information

S1 File Survey on residents’ expectations, sense of place, and perceived value in rural tourism: A generational perspective. (DOCX)

S2 File Inclusivity in global research. (DOCX)

S3 Data Dataset. Raw survey data.
(XLSX)

S4 Data Dataset. Variable codebook.
(DOCX)
