# Supplementary material for: When place and generation matter: Understanding resident satisfaction in rural tourism
Source: PLoS One. 2026 Jul 24;21(7):e0353554. doi: 10.1371/journal.pone.0353554 (PMC13399326; doi:10.1371/journal.pone.0353554)
Supplement: S1 File — (DOCX) [file pone.0353554.s001.docx]

**Survey on Residents’ Expectations, Sense of Place, and Perceived Value in Rural Tourism: A Generational Perspective**

**PART A:** **DEMOGRAPHIC CHRATICRISTICS**

| 1. **Gender** | 1. Male 2. Female |
| --- | --- |
| 1. **Education**   （Level of Education Received and Obtained） | 1. Elementary or lower 2. Middle school 3. High school or secondary school 4. University or post-secondary school 5. Postgraduate 6. Doctorate students |
| 1. **Age** | 1. 17 years or younger 2. 18-29 years 3. 30-39 years 4. 40-49 years 5. 50-59 years 6. 60 years or older (__________ years old) |
| 1. **Your Monthly income (Chinese RMB)** | 1. Less than 1,500 Yuan 2. 1,501 - 3,000 Yuan 3. 3,001 - 4,500 Yuan 4. 4,501 – 6,000 Yuan 5. 6,001 – 7,500 Yuan 6. 7,501 – 9,000 Yuan 7. 9,001 – 10,500 Yuan 8. More than 10,501 Yuan 9. Others (_____________Yuan) |
| 1. **General frequency of travel** | 1. Frequently 2. Rarely 3. Never |
| 1. **Sources of Information** | 1. Travel agency 2. Word of mouth 3. Internet 4. Travel brochure 5. Others (__________________________) |

**PART B: RESEARCH VARIABLES**

| 1. Expectations | **Strongly Disagree** | **1 to 5** | | | **Strongly**  **Agree** |
| --- | --- | --- | --- | --- | --- |
|  | **1** | **2** | **3** | **4** | **5** |
| 1. I expect to see stunning scenery. |  |  |  |  |  |
| 1. I hope to witness the historical and cultural legacy. |  |  |  |  |  |
| 1. I wish to experience comfortable and safe rural environments. |  |  |  |  |  |
| 1. I expect hospitality and local friendliness. |  |  |  |  |  |
| 1. Place attachment | **Strongly Disagree** | **1 to 5** | | | **Strongly**  **Agree** |
|  | **1** | **2** | **3** | **4** | **5** |
| 1. I am happy to visit traditional village cultural landscape of this area. |  |  |  |  |  |
| 1. I would like to visit this traditional village cultural landscape for a long time. |  |  |  |  |  |
| 1. I enjoy this traditional village cultural landscape more than any other village cultural landscape. |  |  |  |  |  |
| 1. I feel a strong attachment to this traditional village cultural landscape. |  |  |  |  |  |
| 1. Place identity | **Strongly Disagree** | **1 to 5** | | | **Strongly**  **Agree** |
|  | **1** | **2** | **3** | **4** | **5** |
| 1. I feel connected to the traditional village cultural landscape in this area. |  |  |  |  |  |
| 1. I have a strong identification with the cultural landscape of this traditional village. |  |  |  |  |  |
| 1. I perceive the cultural landscape of this traditional village as an integral part of my identity. |  |  |  |  |  |
| 1. The cultural landscape of this traditional village is very special to me. |  |  |  |  |  |
| 1. Emotional value | **Strongly Disagree** | **1 to 5** | | | **Strongly**  **Agree** |
|  | **1** | **2** | **3** | **4** | **5** |
| 1. I enjoy the traditional village cultural landscape of this area. |  |  |  |  |  |
| 1. Traditional village cultural landscape in this area gives me a sense of pleasure. |  |  |  |  |  |
| 1. I feel a sense of relaxation in this traditional village cultural landscape. |  |  |  |  |  |
| 1. Traditional village cultural landscapes in this area make me feel good. |  |  |  |  |  |
| 1. The architectural styles of buildings in this traditional village attract me. |  |  |  |  |  |
| 1. Functional Value | **Strongly Disagree** | **1 to 5** | | | **Strongly**  **Agree** |
|  | **1** | **2** | **3** | **4** | **5** |
| 1. Traditional village cultural landscapes in this area consistently maintain a high quality. |  |  |  |  |  |
| 1. Traditional village cultural landscapes in this area are well made. |  |  |  |  |  |
| 1. The traditional village cultural landscape in this area meets acceptable quality standards. |  |  |  |  |  |
| 1. This traditional village cultural landscape is very dependable. |  |  |  |  |  |
| 1. Overall Satisfaction: Please rate your satisfaction on the following items | **Strongly Dissatisfied** | **1 to 5** | | | **Strongly**  **Satisfied** |
|  | **1** | **2** | **3** | **4** | **5** |
| 1. Overall, how satisfied are you with the traditional village cultural landscapes in this area? |  |  |  |  |  |
| 1. How satisfied are you with this traditional village cultural landscapes compared to your expectations? |  |  |  |  |  |
| 1. Considering the time and effort you invested, how satisfied are you with the traditional village cultural landscapes in this area? |  |  |  |  |  |
| 1. How satisfied and happy are you with your decision to visit traditional village cultural landscapes? |  |  |  |  |  |
