# Supplementary material for: When place and generation matter: Understanding resident satisfaction in rural tourism
Source: PLoS One. 2026 Jul 24;21(7):e0353554. doi: 10.1371/journal.pone.0353554 (PMC13399326; doi:10.1371/journal.pone.0353554)
Supplement: S4 Data — Variable codebook. (DOCX) [file pone.0353554.s004.docx]

| **Variable** | **Description** |
| --- | --- |
| Gen | 1 = Gen Z; 2 = Non-Gen Z |
| EX01 | Expectation item 01 |
| EX02 | Expectation item 02 |
| EX03 | Expectation item 03 |
| EX04 | Expectation item 04 |
| PA01 | Place attachment item 01 |
| PA02 | Place attachment item 02 |
| PA03 | Place attachment item 03 |
| PA04 | Place attachment item 04 |
| PI01 | Place identity Item 01 |
| PI02 | Place identity Item 02 |
| PI03 | Place identity Item 03 |
| PI04 | Place identity Item 04 |
| EV01 | Emotional value item 01 |
| EV02 | Emotional value item 02 |
| EV03 | Emotional value item 03 |
| EV04 | Emotional value item 04 |
| EV05 | Emotional value item 05 |
| QFV01 | Functional value item 01 |
| QFV02 | Functional value item 02 |
| QFV03 | Functional value item 03 |
| QFV04 | Functional value item 04 |
| SA01 | Satisfaction item 01 |
| SA02 | Satisfaction item 02 |
| SA03 | Satisfaction item 03 |
| SA04 | Satisfaction item 04 |
